# Equine Immunoglobulin and Equine Neutralizing F(ab′)_2_ Protect Mice from West Nile Virus Infection

**DOI:** 10.3390/v8120332

**Published:** 2016-12-18

**Authors:** Jiannan Cui, Yongkun Zhao, Hualei Wang, Boning Qiu, Zengguo Cao, Qian Li, Yanbo Zhang, Feihu Yan, Hongli Jin, Tiecheng Wang, Weiyang Sun, Na Feng, Yuwei Gao, Jing Sun, Yanqun Wang, Stanley Perlman, Jincun Zhao, Songtao Yang, Xianzhu Xia

**Affiliations:** 1 College of Veterinary Medicine, Northeast Agricultural University, Harbin 150030, China; cjn052015@163.com; 2 Institute of Military Veterinary, Academy of Military Medical Sciences, Changchun 130122, China; zhaoyongkun1976@126.com (Y.Z.); wangh25@hotmail.com (H.W.); CC_xiaocao9ye@163.com (Z.C.); linglong662222@163.com (Q.L.); m13894809942@163.com (Y.Z.); yanfeihu62041@126.com (F.Y.); wgcha@163.com (T.W.); sunweiyang1987@163.com (W.S.); fengna0308@126.com (N.F.); gaoyuwei@gmail.com (Y.G.); 3 Jiangsu Co-innovation Center for Prevention and Control of Important Animal Infectious Disease and Zoonoses, Yangzhou 225009, China; 4 College of Veterinary Medicine, Jilin University, Changchun 130062, China; zerg1230@163.com (B.Q.); jin8616771@163.com (H.J.); 5 State Key Laboratory of Respiratory Diseases, Guangzhou Institute of Respiratory Disease, The First Affiliated Hospital of Guangzhou Medical University, Guangzhou 510120, China; sj-ji@163.com (J.S.); wangyanqun2008@163.com (Y.W.); 6 Department of Microbiology, University of Iowa, Iowa City, IA 52242, USA; stanley-perlman@uiowa.edu

**Keywords:** West Nile virus, equine immunoglobulin, F(ab′)_2_ fragments, mice

## Abstract

West Nile virus (WNV) is prevalent in Africa, Europe, the Middle East, West Asia, and North America, and causes epidemic encephalitis. To date, no effective therapy for WNV infection has been developed; therefore, there is urgent need to find an efficient method to prevent WNV disease. In this study, we prepared and evaluated the protective efficacy of immune serum IgG and pepsin-digested F(ab′)_2_ fragments from horses immunized with the WNV virus-like particles (VLP) expressing the WNV M and E proteins. Immune equine F(ab′)_2_ fragments and immune horse sera efficiently neutralized WNV infection in tissue culture. The passive transfer of equine immune antibodies significantly accelerated the virus clearance in the spleens and brains of WNV infected mice, and reduced mortality. Thus, equine immunoglobulin or equine neutralizing F(ab′)_2_ passive immunotherapy is a potential strategy for the prophylactic or therapeutic treatment of patients infected with WNV.

## 1. Introduction

In recent years, the incidences of West Nile virus (WNV) infection from Culex to humans have increased greatly due to global travel, which raises the public health concern that the 1999–2002 WNV pandemic in North America could recur [1,2]. Some vaccines and treatments have been developed, including imino sugar derivatives [3], DNA vaccines [4], interferon treatment, and human monoclonal antibodies [5]. The clinical evaluation of hE16 and other mAb therapeutics for flavivirus infections should monitor for the selection or emergence of resistant variants, especially in immunocompromised patients who are a likely target population for the treatment of severe WNV infections [6]. The use of a large dose of ribavirin inhibited WNV replication in vitro [7]; however, it was less efficient in vivo—the mortality of the hamsters infected with WNV increased after the ribavirin injection [8]. The WNV RNA replication process is inhibited in vitro by mycophenolate (another candidate inhibitor) [9], which showed no protective effect in vivo. Another clinical study indicated that interferon alpha (IFN-α) could be protective against WNV infection [10]; however, only limited evidence has shown that IFN-α could cure patients with serious WNV encephalitis.

Several WNV vaccines have been recently developed, including inactivated, subunit, and live attenuated vaccines [11]. However, vaccine immunizations might not protect patients who have already developed WNV neuroinvasion. As of now, there are no licensed vaccines or therapeutics for human use. In the study of Engle and Diamond, they found that immune mouse antibodies cannot be directly used in humans due to the heterogeneity of intact mouse IgG, which has the risk of inducing an allergic response in humans; human IgGs are not readily available in large quantities [12]. Passive immunotherapy has been proven useful for many infectious diseases. The administration of polyclonal immunoglobulins from hyperimmune animals or humans has controlled hepatitis B virus (HBV) [13] and human immunodeficiency virus (HIV) [14]. The equine antibodies—especially the F(ab′)2 fragments with reduced heterogeneity—are polyclonal, easy to purify in large quantities, and relatively inexpensive and successfully used to prevent Rabies virus infection [15] and snake venom in humans [16,17]. They can serve as a useful preparedness for WNV pandemic.

Here, we prepared serum IgG from horses immunized with WNV virus-like particles (VLP). To avoid potential allergic responses, pepsin was used to digest the antibodies and to generate the F(ab′)2 fragments. The protective efficacy of the serum IgG and F(ab′)2 fragments against WNV was evaluated in vitro and in vivo. The serum IgG and F(ab′)2 fragments neutralized WNV infection in tissue culture, as determined by a plaque reduction assay. Furthermore, prophylactic and therapeutic treatment of mice with purified IgG or F(ab′)2 fragments significantly decreased the viral loads in the spleens and brains of WNV-infected mice, and reduced mortality. These results indicate that equine immunoglobulin and equine neutralizing F(ab′)2 passive immunotherapy could be potentially useful for the treatment of patients infected with WNV.

## 2. Materials and Methods

### 2.1. Antigen Preparation

Sf9 cells were infected with recombinant baculoviruses co-expressing the WNV structural proteins M and E at a multiplicity of infection (MOI) of 0.5. The culture supernatant was harvested 72 h post infection, and was centrifuged at 5000 rpm for 20 min to remove the cell debris. The supernatant was ultra-centrifuged at 38,000 rpm for 1.5 h at 4 °C. The VLP pellets were resuspended in phosphate-buffered saline (PBS) and loaded on a 10%–30%–50% discontinuous sucrose gradient. After 1.5 h of ultra-centrifugation at 30,000 rpm at 4 °C, the WNV-VLP-containing bands between the 10%–30% sucrose were collected.

### 2.2. Inoculation of Horses

Two 4–6-year-old healthy brown horses (300–350 kg in weight) that had no detectable antibodies against WNV were provided by the Military Hongshan Stud Farm (Changchun, China), and were intramuscularly multi-point injected in the submandibular region and backside with 0.5, 1.5, 2.0, 3.0, and 5.0 mg of WNV-VLP with Freund’s adjuvant (complete/incomplete) (Sigma, St. Louis, MO, USA) on days 0, 7, 14, 28, and 42 (5 times), respectively. The sera were collected from the jugular vein 2 weeks after each immunization, and were stored at −20 °C for further analysis.

### 2.3. WNV-Specific Antibody Measurement

WNV-specific antibodies in the serum were measured by an indirect enzyme-linked immunosorbent assay (ELISA) using purified WNV EDIII (structural domain III of the E protein). The 96-well microtiter plates (Corning Costar, cat# 42592, Corning, NY, USA) used for ELISA were EIA/RIA 1 × 8 Stripwell plates. The featured polystyrene 96-well microtiter plates are flat bottomed and certified for high capacity binding of proteins. Briefly, 96-well microtiter plates were pre-coated with 100 μL of purified EDIII antigen diluted in 0.02 mol/L carbonate sodium buffer (pH 9.6) at a final concentration of 10 μg/mL, and were incubated at 4 °C overnight. After blocking with skim milk for 2 h at 37 °C, 100 μL of two-fold serially diluted serum samples were added to the wells, which were then incubated at 37 °C for 1.5 h. The plate was washed three times with PBS containing 0.5% Tween-20 (PBST), and then 100 μL of an HRP (horseradish peroxidase)-labeled goat antibody against horse IgG (Bios, China) was added (diluted 1:20,000), and the plate was incubated at 37 °C for 1 h. After washing with PBST, 100 μL of the substrate 3,3’,3,5’-tetramethyl benzidine (TMB) (Sigma) was added to each well and incubated for 30 min. The reaction was stopped by adding 50 μL of 0.5 M H_2_SO_4_. The optical density (OD) values were measured using a 96-well ELISA plate reader at a wavelength of 450 nm (Bio-Rad, Los Angeles, CA, USA). The titer results were indicated as the reciprocal of the serum maximum dilution multiple, as the ratio of positive OD value to negative OD value was more than 2.

### 2.4. Immunoglobulin Purification

Horse anti-WNV serum was diluted with equal volumes of saline, and 1/2 volume of a saturated ammonium sulfate solution was then added. The solution was mixed gently at room temperature for 30 min and then centrifuged at 5000 rpm for 20 min. The precipitates were dissolved in saline before 1/3 volume of ammonium sulfate was added. After incubation at room temperature for 30 min, the solution was centrifuged at 5000 rpm for 20 min. The precipitates were dissolved in saline and dialyzed overnight at 4 °C to remove the salt.

### 2.5. Preparation of F(ab′)_2_ Fragments

The pH of the horse anti-WNV serum was adjusted to 3.3 with 1 mol/L HCl. Pepsin was activated by a NaAc solution (pH 3.3) and then added to the diluted horse antiserum and digested at 30 °C for 2.5 h. The reaction was stopped by adjusting the pH to 7.2 with 1 mol/L NaOH. Then, the solution was applied to a Protein-A column, followed by a Protein-G column. The purified protein was subjected to sodium dodecyl sulfate polyacrylamide gel electrophoresis (SDS-PAGE) followed by Coomassie blue staining. The F(ab′)_2_ purity was analyzed with a thin slice scan, and the protein was stored at 4 °C until use.

### 2.6. Mice, Virus, and Cells

*Mavs^−/−^* mice on a C57BL/6 background were initially obtained as a generous gift from S. Akira (Osaka University, Osaka, Japan) [18,19,20]. C57BL/6mice were purchased from Charles River Laboratories. All mice were maintained in the specific-pathogen-free Animal Care Facility at the University of Iowa. All protocols were approved by the University of Iowa Institutional Animal Care and Use Committee. The WNV strain TX 2002-HC (WNV-TX) was propagated as previously described [18]. The working stocks of WNV-TX were generated by a single round of amplification in Vero cells. The titers of virus stocks were determined by a standard plaque assay using Vero cells, as previously described [21]. The Vero cells were maintained in Dulbecco’s modified Eagle medium (DMEM) supplemented with 10% fetal bovine serum (FBS). All work with WNV was conducted in the University of Iowa bio-safety level 3 (BSL 3) laboratory.

### 2.7. WNV Plaque Reduction Neutralization Assay

The serum samples, purified IgG, or F(ab′)_2_ were serially diluted in DMEM and mixed 1:1 with 80 PFU (Plaque Forming Unit) WNV. After incubation at 37 °C for 1 h, the mixture was added to Vero cells, and the plates were incubated at 37 °C in 5% CO_2_ for an additional 1 h. After absorption, the cells were overlaid with 1.2% agarose. After further incubation for 3 days, the agarose plugs were removed. The plaques were visualized by 0.1% crystal violet (contains 2% ethanol and 20% methanol) staining.

### 2.8. Mouse Challenge and Antibody Treatment

Eight to 12-week-old wild type C57BL/6 mice or *Mavs^−/−^* mice were treated with 500 μg of purified horse immune IgG or purified horse immune F(ab′)_2_ via the intraperitoneal (i.p.) route one day before and/or after subcutaneous (s.c.) footpad infection with 100 PFU WNV. The mice were monitored for morbidity and mortality daily. The spleens and brains were harvested at the indicated time points and titered using Vero cells.

## 3. Results

### 3.1. Immunization and Evaluation of Equine Antibodies

**Figure 1:**
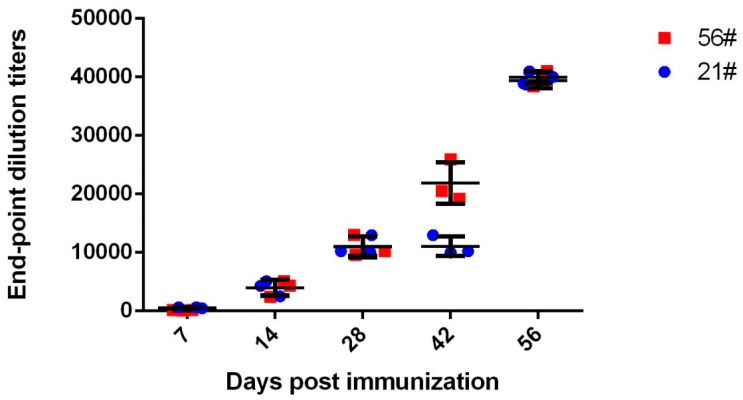
ELISA titers of West Nile virus (WNV) EDIII (structural domain III of the E protein)-specific antibody in immunized horse sera. Horses (*n* = 2, labeled as 21# and 56#) were intramuscularly immunized with WNV-VLP (virus-like particles) and boosted every two weeks for an additional four cycles. The sera were collected 2 weeks after each immunization.

WNV-VLP were produced and purified as previously described [22], and 4–6-year-old healthy horses were intramuscularly immunized with WNV-VLP with Freund’s adjuvant and boosted every 2 weeks for 4 cycles. Serial serum samples were collected. The IgG titers against WNV EDIII were measured by ELISA (Figure 1). The EDIII-specific IgG titers in the serum were all above 1:20,480 after five immunizations (Figure 1).

### 3.2. Generation and Purification of IgG and F(ab′)_2_

**Figure 2:**
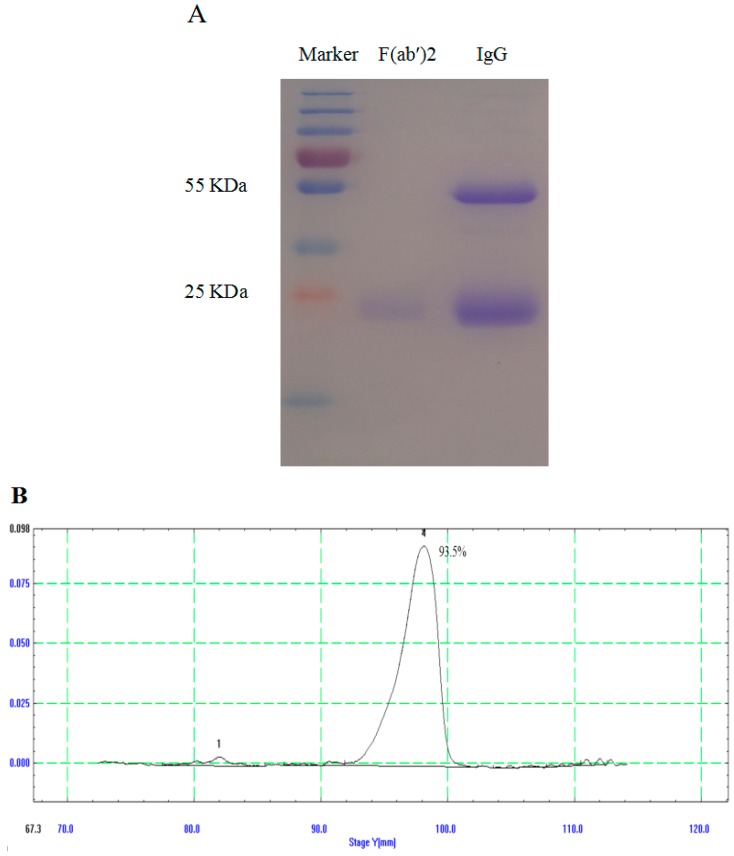
Generation and purification of IgG and F(ab′)_2_. A saturated solution of ammonium sulfate was added to the serum to precipitate the IgG, and F(ab′)_2_ was generated by digesting the IgG with pepsin followed by Protein-A/G chromatography. (**A**) SDS-PAGE and Coomassie blue staining of the purified IgG and F(ab′)_2_; (**B**) The purity of the F(ab′)_2_ fragment was determined by a thin slice scan.

Immunoglobulins were precipitated using saturated ammonium sulfate and digested by pepsin. The F(ab′)_2_ fragments were purified by Protein-A/G chromatography. The integrity of IgG and F(ab′)_2_ fragments was evaluated using SDS-PAGE (Figure 2A). The purity of the F(ab′)_2_ fragments after Protein-A/G chromatography was higher than 93.5% (Figure 2B).

### 3.3. Equine Antibodies Neutralized WNV in Cell Culture

**Figure 3:**
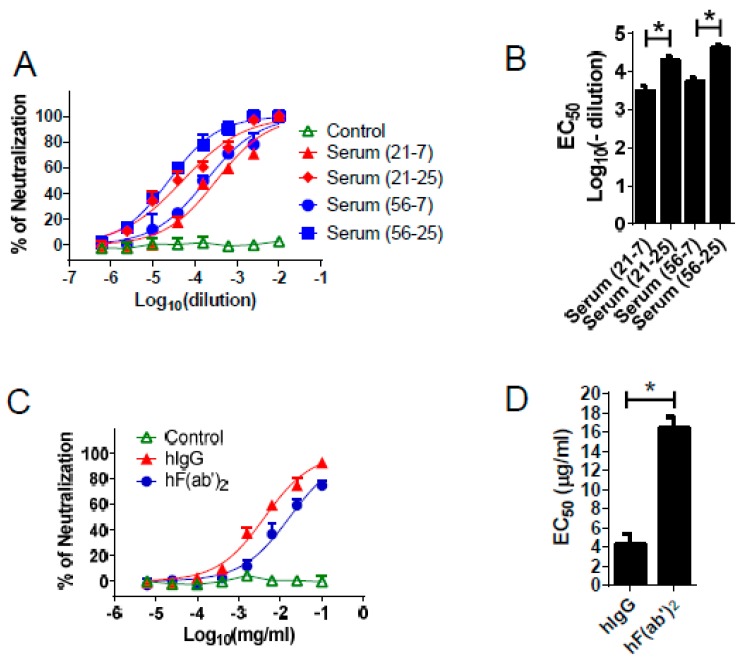
Neutralization assay for WNV using immune horse serum, purified IgG, and F(ab′)_2_ in vitro. The “21” and “56” are the horse ID numbers. The “21–7”, “56–7” and the “21–25”, “56–25” represent the sera that were collected after the fourth or fifth immunization, respectively. (**A**,**B**) Serum or (**C**,**D**) antibody samples from “56–25” were serially diluted in Dulbecco’s modified Eagle medium (DMEM) and mixed 1:1 with 80 PFU (Plaque Forming Unit) WNV. After a 1-h incubation at 37 °C, the mixture was added to Vero cells for an additional 1 h. After absorption, the cells were then overlaid with 1.2% agarose. After a further incubation for 3 days, the agarose plugs were removed. The plaques were visualized by 0.1% crystal violet staining; (**B**) The dilutions; and (**D**) Concentrations for 50% of the maximal neutralizing effect are shown. * *p* < 0.05. Normal horse IgG was used as the control.

Using a plaque reduction-neutralizing assay, we confirmed that the immune sera efficiently neutralized WNV infection in vitro, with half effect maximal dilutions of 10^−4.3^ for the 21–25 serum and 10^−4.6^ for the 56–25 serum (Figure 3A,B). Furthermore, we found that the purified equine IgG and F(ab′)_2_ also neutralized WNV infection with half effective maximal concentrations (EC50) of 4.1 μg/mL and 16.5 μg/mL for the IgG and F(ab′)_2_ fragments, respectively (Figure 3C,D). Of note, the EC50 of the F(ab′)_2_ fragments was lower than that of the IgG in vitro. These results suggest that equine antibody products exhibit highly potent neutralizing activity against WNV in tissue culture.

### 3.4. Passive Transfer of Equine Antibodies Protected WNV-Infected Mice

**Figure 4:**
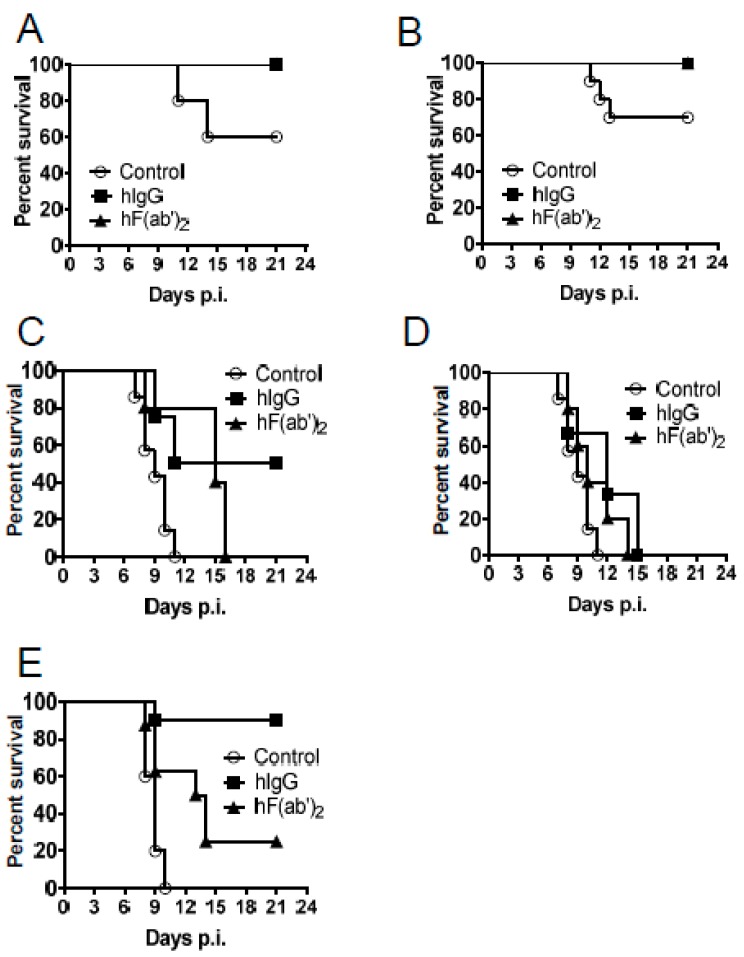
Immune purified IgG and F(ab’)_2_ transfer protected WNV infected mice. Eight to twelve-week-old wild type (WT) C57BL/6 mice (**A**,**B**) or *Mavs^−/−^* mice (**C**–**E**) were infected with 100 PFU WNV in the footpad (s.c.), and 500 μg of the purified horse immune IgG (hIgG) or the purified horse immune F(ab’)_2_ (hF(ab’)_2_) was injected (i.p.) into infected mice one day before (**A**,**C**) or after infection (**B**,**D**), or on both days (**E**). The mice were monitored for morbidity and mortality daily. The mice that received normal horse IgG were used as the controls. (**A**) *n* = 10, Control; *n* = 5, hIgG; *n* = 5, hF(ab′)_2_; (**B**) *n* = 10, Control; *n* = 5, hIgG; *n* = 5, hF(ab′)_2_; (**C**) *n* = 7, Control; *n* = 8, hIgG; *n* = 5, hF(ab′)_2_; (**D**) *n* = 7, Control; *n* = 6, hIgG; *n* = 5, hF(ab′)_2_; (**E**) *n* = 5, Control; *n* = 10, hIgG; *n* = 8, hF(ab′)_2_. For Figure 4C, *p* = 0.009 when Control group compared to hIgG group. *p* = 0.0123 when Control group compared to F(ab′)_2_ group. For Figure 4E, *p* = 0.0009 when Control group compared to hIgG group. *p* = 0.0291 when Control group compared to F(ab′)_2_ group.

**Figure 5:**
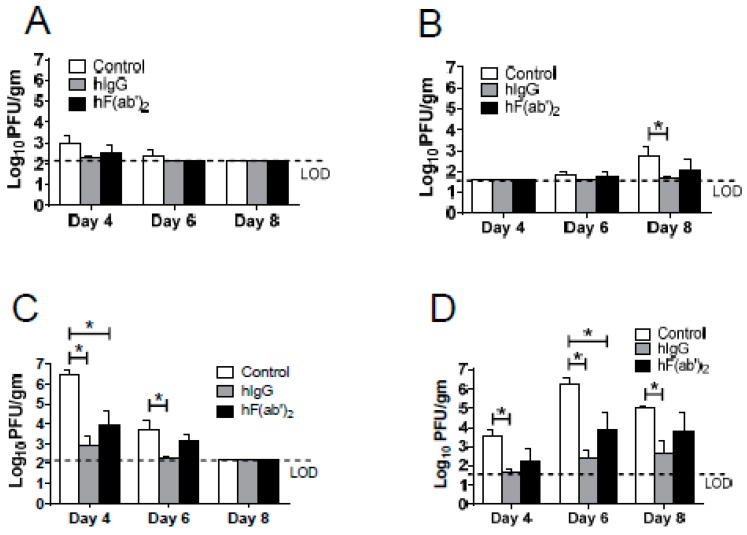
Immune purified IgG and F(ab′)_2_ transfer reduced the WNV load in the mice. Eight to twelve-week-old WT (**A**,**B**) and *Mavs^−/−^* mice; (**C**,**D**) were infected with 100 PFU WNV in the footpad (s.c.), and 500 μg of the purified horse immune IgG (hIgG) or the purified horse immune F(ab′)_2_ (hF(ab′)_2_) was transferred (i.p.) one day before and one day after infection. To obtain the virus titers, the spleens (**A**,**C**) and brains (**B**,**D**) were homogenized at the indicated time points and titered using Vero cells. The titers are expressed as PFU/g tissue. *n* = 3–4 mice/group/time point. * *p* < 0.05. The mice that received normal horse IgG were used as the controls.

To validate the protective effect of the equine antibodies in vivo, we transferred purified horse immune IgG or purified horse immune F(ab′)_2_ intraperitoneally into mice one day before or after infection, or on both days. After WNV infection, all wild type (WT) mice receiving the immune IgG or F(ab′)_2_ survived, whereas 30%–40% of WT mice receiving the control horse IgG died, regardless of whether the antibodies were administered before or after the infection. The mitochondrial antiviral-signaling protein (MAVS) is a key adaptor molecule in the RIG (Retinoic Acid-inducible Gene)-I-like receptor RNA-sensing pathway [23]; *Mavs^−/−^* mice were highly susceptible and uniformly succumbed to WNV infection, with death occurring by 11 days (Figure 4C–E) [18]. However, 50% of the *Mavs^−/−^* mice that received the horse IgG one day before the infection survived. In contrast, in the in vitro neutralizing assays (Figure 3C,D), the mice that received the F(ab′)_2_ fragments showed delayed mortality; however, 100% of these mice died within 15 days (Figure 4C). An immune antibody transfer one day after the infection was not protective in the *Mavs^−/−^* mice (Figure 4D). Furthermore, to determine whether two doses of the equine antibody treatment could enhance the prophylactic function in *Mavs^−/−^* mice, we treated mice both one day before and one day after WNV infection. All *Mavs^−/−^* mice from the control group died, whereas 90% of mice that received the horse immune IgG survived; the F(ab′)_2_ treatment protected only 30% of the mice. Next, we asked whether a passive transfer of the equine immune antibodies could decrease the WNV load in vivo. WT and *Mavs^−/−^* mice were infected with WNV at the indicated time points, and the spleens and brains were harvested for titers. As expected, antibody treatment decreased the virus load in both the WT and *Mavs^−/−^* mice (Figure 5).

## 4. Discussion

Previous studies have shown that the WNV is spread by air transportation or the migration of birds [24]. Currently, WNV is circulating in not only North America but also Russia, India, and Australia [24]. No effective treatment or licensed vaccine exists that has been proven for human use.

In this study, we demonstrated that EDIII-specific IgG titers in the serum were up to 1:40,960 after five immunizations, and we also verified that the purity of the F(ab′)2 fragments after Protein-A/G chromatography was higher than 93.5%. Then, we found that the equine antibody products exhibit highly potent neutralizing activity against WNV in tissue culture. Importantly, we confirmed that equine anti-WNV immunoglobulin and neutralizing F(ab′)2 decreased the virus load and protected mice from a lethal WNV infection.

We used C57BL/6 mice and *Mavs^−/−^* mice on C57BL/6 background for this study. C57BL/6 mice are immunocompetent and are broadly used in WNV studies with a mortality rate of around 20%–40%. *Mavs^−/−^* mice are immunocompromised with the deficiency of MAVS gene which is important for viral RNA sensing. These mice were uniformly succumbed to WNV infection. These models perfectly mimic WNV infection in humans. In human infections, most of infected patients with intact immune system get mild disease, while the immunocompromised patients and the elderly tend to develop severe neuroinvasion.

WNV-VLP were used to immunize healthy horses and generate the equine anti-WNV IgG and F(ab′)2. The results showed that it is technically feasible to rapidly generate large amounts of equine neutralizing antibodies against WNV. The F(ab′)_2_ fragments help alleviate immune hypersensitivity, making the antibodies safer for human use. More importantly, the equine anti-WNV antibodies are polyclonal and can recognize more antigen determinants on WNV glycoproteins and membranes than a monoclonal antibody, which could potentially avoid antibody escape.

Highly purified equine F(ab′)2 fragments have been shown to be safe in humans and have been used to treat many diseases for decades. Equine F(ab′)2 fragments surmount the bottleneck of antibody yield, possess broader antigenic coverage, and avoid the emergence of escape mutants [25,26,27]. Of note, equine antibodies are relatively economical and are readily available. Although convalescent plasma from patients has shown some protective efficacy, the neutralizing antibody titers in patients are generally low, and the limited number of survivors makes this approach impractical [28,29,30].

The quality of the neutralization antibody can influence the treatment efficacy, which may depend on the quantity and quality of the F(ab′)2 fragments. According to our assays, the purity of the F(ab′)2 fragments was 93.5%, which met the requirement of the bio-preparation in China. The results of this study show that F(ab′)2 fragments are not as efficient as IgG, both in vitro and in vivo, and a possible factor contributing to this outcome is that F(ab′)2 has a shorter half-life than IgG.

In summary, by immunizing healthy horses with WNV-VLP, we successfully developed the first equine IgG-derived F(ab′)2 fragments that could neutralize WNV both in vitro and vivo. Both prophylactic and therapeutic treatments with equine anti-WNV immunoglobulin or neutralizing F(ab′)2 decreased the viral loads in the spleens and brains, and protected mice from a lethal WNV infection. Therefore, horses immunized with WNV-VLP could serve as a useful initial source for developing protective F(ab′)2 fragments, which may be useful for preparedness and serve as a strategic reserve for a potential WNV epidemic and other emergent pathogens.

## 5. Conclusions

We successfully developed the first equine IgG-derived F(ab′)_2_ fragments that could neutralize WNV both in vitro and vivo. Both prophylactic and therapeutic treatments with equine anti-WNV immunoglobulin or neutralizing F(ab′)_2_ decreased the viral loads in the spleens and brains, and protected mice from a lethal WNV infection. These results indicate that equine immunoglobulin and equine neutralizing F(ab′)_2_ passive immunotherapy could be potentially useful for the treatment of patients infected with WNV.
